# Supplementary material for: ACP-MHCNN: an accurate multi-headed deep-convolutional neural network to predict anticancer peptides
Source: Sci Rep. 2021 Dec 8;11:23676. doi: 10.1038/s41598-021-02703-3 (PMC8654959; doi:10.1038/s41598-021-02703-3)
Supplement: Supplementary file 1 — Supplementary Information. [file 41598_2021_2703_MOESM1_ESM.docx]

Table S1: Generalization testing for ACP-DL

| Dataset | Accuracy | Sensitivity | Specificity | Precision | MCC |
| --- | --- | --- | --- | --- | --- |
| Trained on ACP-740 and  Tested on ACP-240 | 62.0 | 67.4 | 63.5 | 63.5 | 0.23 |
| Trained on ACP-740 and  Tested on ACP-164 | 67.0 | 85.3 | 48.7 | 62.5 | 0.37 |
